# Connexin Composition in Apposed Gap Junction Hemiplaques Revealed by Matched Double-Replica Freeze-Fracture Replica Immunogold Labeling

**DOI:** 10.1007/s00232-012-9454-2

**Published:** 2012-07-04

**Authors:** John E. Rash, Naomi Kamasawa, Kimberly G. V. Davidson, Thomas Yasumura, Alberto E. Pereda, James I. Nagy

**Affiliations:** 1 Department of Biomedical Sciences, Colorado State University, Fort Collins, CO 80523 USA; 2 Program in Molecular, Cellular, and Integrative Neurosciences, Colorado State University, Fort Collins, CO 80523 USA; 3 Electron Microscopy Core Facility, Max Planck Florida Institute, Jupiter, FL 33458 USA; 4 Dominick P. Purpura Department of Neuroscience, Albert Einstein College of Medicine, Bronx, NY 10461 USA; 5 Department of Physiology, Faculty of Medicine, University of Manitoba, Winnipeg, MB, R3E 0J9 Canada

**Keywords:** Astrocyte, Ependymocyte, Glia, Neuron, Oligodendrocyte

## Abstract

Despite the combination of light-microscopic immunocytochemistry, histochemical mRNA detection techniques and protein reporter systems, progress in identifying the protein composition of neuronal versus glial gap junctions, determination of the differential localization of their constituent connexin proteins in two apposing membranes and understanding human neurological diseases caused by connexin mutations has been problematic due to ambiguities introduced in the cellular and subcellular assignment of connexins. Misassignments occurred primarily because membranes and their constituent proteins are below the limit of resolution of light microscopic imaging techniques. Currently, only serial thin-section transmission electron microscopy and freeze-fracture replica immunogold labeling have sufficient resolution to assign connexin proteins to either or both sides of gap junction plaques. However, freeze-fracture replica immunogold labeling has been limited because conventional freeze fracturing allows retrieval of only one of the two membrane fracture faces within a gap junction, making it difficult to identify connexin coupling partners in hemiplaques removed by fracturing. We now summarize progress in ascertaining the connexin composition of two coupled hemiplaques using matched double-replicas that are labeled simultaneously for multiple connexins. This approach allows unambiguous identification of connexins and determination of the membrane “sidedness” and the identities of connexin coupling partners in homotypic and heterotypic gap junctions of vertebrate neurons.

## Introduction and Historical Perspective

Vertebrate gap junctions consist of connexin proteins that assemble as hexamers to form connexon “hemichannels” that link across the extracellular space, forming leakless channels that permit the direct intercellular transport of water, ions and small molecules [≤450 Da (Hu and Dahl 1999)]. Of the 20 or 21 connexins expressed in mammals—named according to their molecular weight, measured in kilodaltons (Willecke et al. 2002)—more than half are expressed by cells in the central nervous system (CNS). Due to the cellular heterogeneity and morphological complexity of CNS tissue, assignment of connexin expression in, and understanding the formation of gap junctions between, particular cell types has been problematic. Yet, this has become an important issue, especially in recent years by virtue of the identification of several diseases with major neurological damage caused by mutations in connexins expressed in neural tissues. These include X-linked Charcot–Marie–Tooth disease (CMTX) (Bergoffen et al. 1993), resulting from mutations of Cx32; Pelizaeus–Merzbacher-like disease (PMLD), resulting from mutations of Cx47 (Tress et al. 2011; Uhlenberg et al. 2004), oculodentodigital dysplasia (ODDD), resulting from mutations of Cx43 (Paznekas et al. 2003); keratitis–ichthyosis–deafness syndrome, resulting from mutations of Cx26 (Kelsell et al. 1997; Melchionda et al. 2005); and childhood-onset myoclonic epilepsy, resulting from mutations of Cx36 in the noncoding region (Hempelmann et al. 2006; Mas et al. 2004). Understanding how these connexin mutations impact on physiological processes in the CNS and cause severe debilitating disease requires firm knowledge of the cell types expressing the mutated connexins, the subcellular and histological locations at which gap junctions may be disrupted by these abnormal connexins and the nature of the connexin coupling partners normally occurring at those locations. This knowledge acquired in studies of the glial connexins, as well as in studies of defective ion channels and water-transport pathways used for long-distance potassium siphoning and CNS water homeostasis, led to our formulation of the “gateway hypothesis” (Davidson and Rash 2011; Rash 2010) as a working paradigm for a generalized mechanism underlying the physiological and morphological aberrations found in CMTX, ODDD, PMLD, neuromyelitis optica, Alexander disease (van der Knaap et al. 2001) and other “leukodystrophies” (white matter diseases). However, details of the molecular organization of glial gap junctions with their five gap junction-forming connexins, as well as of gap junctions forming electrical synapses in the CNS, is a work in progress. Here, we outline some outstanding difficulties and present a new approach that may help to resolve some of the existing limitations.

## Connexon Coupling Patterns

**Fig. 1:**
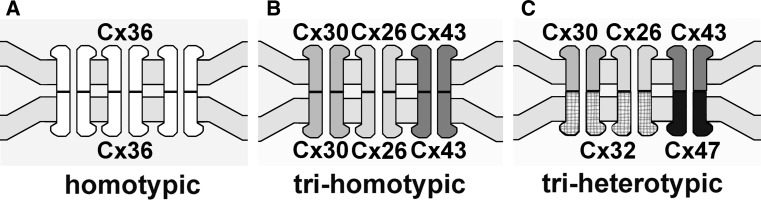
Generalized models of different connexin coupling patterns in the most common type of gap junctions in the CNS. **a** “Homotypic” neuronal gap junction, with intercellular channels composed of Cx36 coupling with Cx36. **b** “Trihomotypic” astrocyte-to-astrocyte gap junction, with intercellular channels composed of Cx43 coupling with Cx43, Cx30 coupling with Cx30 and Cx26 coupling with Cx26. **c** “Triheterotypic” astrocyte-to-oligodendrocyte gap junction, with astrocyte Cx43 coupling with oligodendrocyte Cx47, astrocyte Cx30 coupling with oligodendrocyte Cx32 and astrocyte Cx26 coupling with oligodendrocyte Cx32. Additional permissive coupling pairs are discussed in the text

Individual cell types express from one to four different connexins, allowing for the potential formation of “heteromeric” connexons composed of two or more connexins, for which evidence has been obtained in only a few tissues in vivo (Jiang and Goodenough 1996; Sosinsky 1995). Some neurons express only a single connexin isoform (e.g., most express only Cx36), and these often form gap junctions with other neurons singly expressing the same connexin, thereby forming “homomeric” connexins in both cells that link to form “homotypic” intercellular channels (Fig. 1a, showing the simplest type of neuron-to-neuron gap junction, composed of Cx36, only). However, multiple connexin isoforms are expressed in many types of cells, providing for the possible formation of “bihomotypic” and “trihomotypic” gap junctions. For example, astrocyte-to-astrocyte (A:A) gap junctions usually consist of two or three types of homotypic channels, forming, as one example, a Cx43:Cx43 plus Cx30:Cx30 plus Cx26:Cx26 trihomotypic gap junction (Fig. 1b). In addition, when astrocytes (A) couple to oligodendrocytes (O), which express two other gap junction-forming connexins, Cx47 and Cx32, these “heterologous” A:O gap junctions, are necessarily “bi-” or “triheterotypic” (or even multiheterotypic), composed of several combinations of “permissive” coupling partners, three of which are illustrated in Fig. 1c. [Cx29 is also expressed in oligodendrocytes but does not form gap junctions (Altevogt and Paul 2004).] In A:O junctions, a distinct set of connexins on the astrocyte side (i.e., Cx26, Cx30, Cx43) link with a different set of connexins on the oligodendrocyte side (i.e., Cx32, Cx47) (Altevogt et al. 2002; Altevogt and Paul 2004; Kleopa et al. 2004; Li et al. 1997; Nagy et al. 2004; Nagy and Rash 2000; Scherer et al. 1995). Recent studies of coupling permissiveness in N2A cells expressing glial connexins indicate that, in addition to permissive homotypic combinations (e.g., Cx30/Cx30, Cx43/Cx43, Cx32/Cx32 and Cx47/Cx47), functional coupling can occur between Cx30 and Cx32 and between Cx43 and Cx47 (Orthmann-Murphy et al. 2007), as well as between Cx30 and Cx47 (Magnotti et al. 2011). Finally, homologous cell types (two neurons, for example) expressing two different connexins at the same gap junction plaque may also form either bihomotypic or heterotypic gap junctions. These several coupling configurations may provide vertebrate gap junctions with the molecular basis for attaining functional diversity, including electrical rectification (directionality of current flow), which has been proposed to require molecular asymmetry of apposed connexon hemichannels (Barrio et al. 1991; Palacios-Prado and Bukauskas 2009; Rubin et al. 1992; Verselis et al. 1994). Currently, these functionally distinct configurations of connexins within apposing hemiplaques can be distinguished in vivo only by the double-replica immunogold labeling technique, as described below.

## Sources of Ambiguity in Previous Approaches

### False-Positive Identification of Protein Expression in Neural Cells

False-positive identifications of connexin protein expression in cells and misassignment of those connexins to an inappropriate cell type can occur for three main reasons. The first is inadequate confirmation of anticonnexin antibody specificity, which can result in failure to recognize off-target labeling of proteins. The use of connexin knockout (KO) mice can be considered the “gold standard” for confirmation of specificity. Many of the antibodies that we use have been characterized for specificity by comparison of immunofluorescence and/or immunoblotting results in wild-type mice vs. mice with KO of the various connexins. For connexins relevant here, such characterization has included antibodies against Cx26 (Nagy et al. 2011), Cx30 (Lynn et al. 2011), Cx32 (Nagy et al. 2003), Cx36 (Li et al. 2004), Cx47 (Li et al. 2008b) and Cx57 (Ciolofan et al. 2007). In addition, where possible, we have included the use of two different antibodies generated against different sequences within individual connexins, as well as the use of both rabbit polyclonal and mouse monoclonal antibodies generated against the same sequence in some of the connexins. The specificity of antibodies against Cx43 and Cx45 has been established ultrastructurally by showing their detection in gap junction plaques in identified cell types (Rash et al. 2001).

A second source of error derives from the failure to detect a protein target even when using antibodies with proven specificity and proven immunohistochemical applications. This can arise mainly from nonoptimal tissue preparation for immunostaining, particularly if prescribed fixation protocols are not followed. We have previously emphasized that immunofluorescence detection of some connexins requires very weak fixation conditions, where overfixation results in reduced detection or abolition of immunolabeling entirely (Li et al. 2008b).

**Fig. 2:**
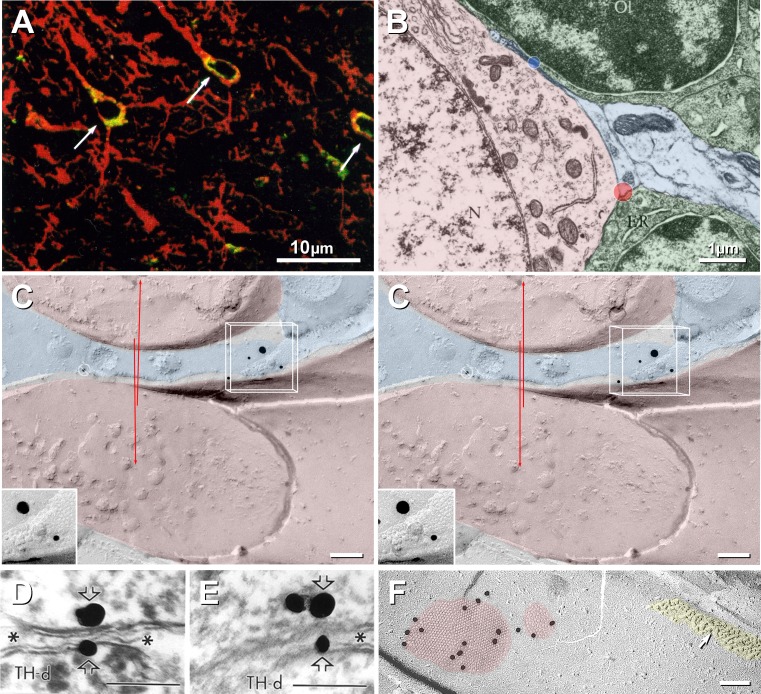
Comparison of limits of resolution of light microscopy (**a**) with ultrastructural resolution (**b**, **c**). **a** Neurons double-stained for Cx43 (*green fluorescence*) and the neuronal marker MAP-2 (*red fluorescence*) but without visualization of intervening glial cells. Without companion bright-field or differential interference optics to reveal other cell types, it is not possible to assign Cx43 unambiguously to the visualized cells, regardless of apparent close proximity. This deficiency is implicit in representative thin-section TEM images. **b** Modified from Peters et al. (1991). The limits of resolution in the blue and red wavelengths are indicated by superimposed* red* and* blue discs*, each of which overlaps cell margins of all three cell types, as well as multiple cytoplasmic membranes. **c** Two neuronal dendritic processes (*red overlays*), with a gap junction linking two thin intervening astrocyte processes (*blue overlays*). The astrocyte gap junction (shown at higher magnification in the *inset*) is double-labeled for Cx26 (12 nm gold) and Cx30 (20 nm gold). The limits of resolution in the *x*, *y* and *z* axes are indicated by the inscribed *three-dimensional box*, which corresponds to a single voxel (volume pixel) at the limit of resolution of confocal LM. If this region had been visualized by LM, with neurons stained red, astrocytes and oligodendrocytes not stained and connexins visualized using green fluorescence (as in **a**), Cx26 and Cx30 would have appeared to be localized to the decussating, small-diameter neuronal processes. *Crossing red arrows* indicate the limit of LM resolution in the red wavelength, suggesting that these two neuronal processes would have been in direct contact, with no room for intervening astrocyte processes. Barred Circle = gold bead on top of replica, as "noise" (Rash and Yasumura 1999). **d**, **e** “Serial sections in which gold–silver labeling for Cx32 (*straight open arrows*) was identified on the cytoplasmic surface of a peroxidase-labeled TH dendrite and in an apposed glial process (*asterisks*) that separates two TH-positive dendrites from one another” (Alvarez-Maubecin et al. 2000). However, we note that Cx32 is an oligodendrocyte connexin and is not found in astrocytes, nor has it been detected in ultrastructurally defined neuronal gap junctions, so we consider these images to represent background “noise” on two nonserial sections, each showing an astrocyte process between two different sets of TH neurons. *Calibration bars* 0.2 μm. **f** Comparison FRIL image of two neuronal gap junctions (*red overlays*) in adult rat retina that were immunogold-labeled for Cx36 (13- and three 20-nm gold beads). Unlabeled glutamate receptor postsynaptic density (*yellow overlay*). Modified from Rash et al. (2001). *Calibration bars* 0.1 μm, unless otherwise indicated

The third source of connexin misassignment arises from the limited resolution of light microscopy (LM). Because of inherent limits of LM resolution, current immunocytochemical methods applied to complex CNS tissues are unable to discern whether specific connexins, reportedly identified either by diffuse cytoplasmic staining (Colwell 2000) or by the presence of both punctate immunolabeling for connexins and widespread cell-surface immunofluorescence (Nadarajah et al. 1996, 1997), link either neurons or glia or both. When only a single cell type (neuron) is visualized by immunofluorescence in CNS tissue, without companion bright-field or differential interference optics to reveal glial cells (Fig. 2a), it is not possible to assign connexins unambiguously to the visualized neuron, regardless of apparent close proximity of connexin labels. This failure to account for CNS tissue complexity is implicit in representative thin-section transmission electron microscopic (TEM) images (Fig. 2b), wherein all spaces between neurons are seen to be completely filled with the two primary types of macroglial cells (astrocytes and oligodendrocytes) found throughout the neuropil and by their even more pervasive thin processes that are also below the limit of LM resolution. For further clarification, the limits of resolution in the blue and red wavelengths are superimposed on the TEM image (Fig. 2b, blue and red discs), revealing that a single pixel at the limit of LM resolution in those wavelengths would overlap multiple plasma membranes of multiple cell types, with the blue dot overlapping with a neuronal plasma membrane, two astrocyte fingers and an oligodendrocyte soma and nucleus. This image suggests that, in the absence of companion ultrastructural examination, complex interdigitations of neuronal and glial processes preclude or make questionable the LM assignment of specific connexins to specific cell types in convoluted CNS tissue. Of course, this problem of assigning proteins to specific cell margins applies equally well to subcellular localization of all other membrane proteins.

To investigate the basis for putative neuronal gap junctions reportedly containing Cx26, Cx32 and Cx43 by freeze-fracture replica immunogold labeling (FRIL) using knife-fractured single replicas (procedure described below) revealed that neuronal processes often had thin astrocyte “fingers” interposed, in this case with a small A:A gap junction labeled for both Cx26 and Cx30 (Fig. 2c; 12 nm gold = Cx26, 20 nm gold = Cx30). The nominal LM limits of resolution in the *x*, *y* and *z* axes are indicated in stereoscopic images by the inscribed three-dimensional box, which corresponds to a single “voxel” (volume pixel) at the resolution limit of confocal LM (0.2 × 0.2 × 0.4 μm in the *x*, *y* and *z* axes, respectively). Moreover, in the red wavelength (which had been used to visualize the margins of the neurons in Fig. 2a), the limit of resolution is ~0.4 μm, or several times the width of the space occupied by the astrocyte fingers (Fig. 2c, crossing red arrows). If this configuration had been imaged by LM in red fluorescence (for neuronal markers) and green (for Cx43, Cx32 or Cx26), the overlay would have appeared to support Cx26 (or any other astrocyte connexin) between the two crossing neuronal processes. Thus, this image graphically demonstrates why ultrastructural approaches are essential for eliminating ambiguities of connexin assignment to specific cell types in CNS tissue.

In addition to those early immunofluorescence reports suggesting that neurons express multiple connexins that are now widely recognized to be “glial” [e.g., Cx26, Cx30, Cx32, Cx43 and Cx47 (Chang et al. 1999; Nadarajah et al. 1996, 1997; Nadarajah and Parnavelas 1999; Teubner et al. 2001; Venance et al. 2000; Zhang et al. 2000)], both Cx32 and Cx26 proteins were reported to occur between neurons at putative gap junctions, as identified by the presence of one to three silver-intensified gold beads at areas where membranes “tended to approach” (Fig. 2d, e); but these areas were not otherwise recognizable as gap junctions, even in these purported “consecutive serial sections.” Unaccountably, the contacting membranes had reversed contour in the successive sections, and the cytoplasm of one section was heavily stained for tyrosine hydroxylase/peroxidase (Fig. 2d), whereas the successive section had little or no staining (Fig. 2e), suggesting that the samples may have been misidentified as representing consecutive serial sections. In contrast, FRIL (Fig. 2f) unambiguously revealed gap junctions as clusters of 10-nm P-face intramembrane particles (IMPs) and/or 9-nm E-face pits (Goodenough and Revel 1970). [P-face = protoplasmic leaflet, E-face = extraplasmic leaflet; established nomenclature defined in Branton et al. (1975).] By FRIL, both neuronal and glial gap junctions were further confirmed by labeling with multiple immunogold beads for appropriate cell type–specific connexins and only in the appropriate ultrastructurally identified cell types (Rash et al. 2001).

To date, no neuronal gap junctions have been detected by FRIL that were immunogold-labeled for any of the consensus “glial” connexins. However, more than 3,000 neuronal gap junctions have been detected that were labeled for Cx36 (Kamasawa et al. 2006; Rash et al. 2005, 2007a, b), and ~100 have been detected in rodent retina that were labeled for Cx45 (Li et al. 2008a) (see the following); but none were labeled for glial connexins, either within neuronal hemiplaques or within the hemiplaques of neuronal coupling partners. This latter observation means that in normal CNS tissues neurons do not couple with glial cells, regardless of the connexins present in each. Presumably, the neuronal connexins are nonpermissive with glial connexins. In contrast, in many of the same double- and triple-labeled samples, many thousands of glial gap junctions were cumulatively labeled by tens of thousands of gold beads for glial connexins, each gold bead representing a separate confirmation of the target connexin in those gap junctions (Nagy et al. 2004; Rash et al. 2001). With no consensus glial connexins ever detected in ultrastructurally identified gap junctions of neurons and with thousands of glial gap junctions labeled for consensus glial connexins and never for neuronal connexins (Nagy et al. 2003, 2004; Nagy and Rash 2000; Rash et al. 2001), it is no longer appropriate to invoke those early reports as evidence for Cx26, Cx30, Cx32, Cx43 or Cx47 in neurons. On the other hand, Cx36 has been identified in sufficient numbers of neuronal gap junctions and in sufficient areas of the CNS to qualify as a reliable immunofluorescence marker for electrical synapses in widespread brain regions [see companion paper (Lynn et al. 2012), this issue].

### False Positives Arising from mRNA Detection Methods

Even when combined with mRNA detection methods (e.g., in situ hybridization, RT-PCR or LacZ reporter methods), detection is common for multiple connexin mRNAs, including multiple glial connexin mRNAs in neurons (Chang et al. 1999; Venance et al. 2000; Zhang et al. 2000; Zhang 2010). In the decade since Fire et al. (1998) first described mRNA suppression by microinterfering RNA (miRNA), it has become clear that most classes of connexin mRNAs appear to be actively prevented from translation into protein by multiple miRNAs, which are particularly abundant in the mammalian brain (Bartel 2004; Berezikov et al. 2006; Farh et al. 2005; Krichevsky et al. 2003; Lim et al. 2005; Miska et al. 2004; Sempere et al. 2004), with some of the “seed matching sequences” for glial vs. neuronal connexins having been identified, consistent with active suppression of glial connexin mRNAs in neurons (Rash et al. 2005). We conclude that in CNS tissues there is such a high incidence of detection of diverse connexin mRNAs without detection of the corresponding connexin protein that such methods have been especially misleading in the identification of the neuronal connexins that are actually expressed (i.e., false correlation of mRNA detection with protein detection). Specifically, one or two neuronal connexin mRNAs but at least five glial connexin mRNAs are routinely detected in neurons by single-cell RT-PCR and by in situ hybridization (Chang et al. 1999; Rash et al. 2005; Venance et al. 2000; Zhang et al. 2000; Zhang 2010). However, none of the five glial connexins that are routinely detected by these mRNA methods appear to be translated into proteins as none were detected by FRIL at ultrastructurally identified gap junctions, even in the same tissues and animal ages as examined by others (Li et al. 2008a; Rash et al. 2005, 2007a, b).

To address these important issues of gap junction connexin composition and the “sidedness” of connexin expression, ultrastructural approaches have been employed, including thin-section TEM (Nagy et al. 1999, 2001; Ochalski et al. 1997; Yamamoto et al. 1990a, b), FRIL and, more recently, double-replica FRIL (DR-FRIL). [FRIL is the abbreviation originally introduced by Gruijters et al. (1987) for “fracture-replica-immunogold labeling” by a method not using the breakthrough SDS detergent washing procedure (Fujimoto 1995, 1997).] Alternative replica labeling procedures and nomenclature include SDS-FRL (Fujimoto 1995, 1997) and double-replica SDS-FRL (Li et al. 2008a). Our version of FRIL originally was distinguished from SDS-FRL by the additional steps of Lexan plastic stabilization of replicas and by confocal grid mapping to the limit of resolution of LM, prior to tissue removal by SDS washing and subsequent immunogold labeling (Rash et al. 1995, 1996). We found the added steps of FRIL to be essential for the analysis of gap junctions in complex CNS tissues. Regardless, we used both DR-SDS-FRL and DR-FRIL methods in this report.

### False Negatives Arising from LacZ mRNA Detection Methods

Data from LacZ reporter systems have been interpreted as showing that a small percentage of neurons in the inner plexiform layer of the retina (i.e., cone bipolar cells) express only Cx45 and not Cx36 (Schubert et al. 2005), whereas the AII amacrine cells to which bipolar cells are known to couple reportedly express only Cx36. This appeared to pose a problem because Cx45 and Cx36 are reported to be “nonpermissive” for forming gap junctions (Teubner et al. 2000). Moreover, three groups using RT-PCR and immunocytochemistry (Han and Massey 2005; Lin et al. 2005) and immunofluorescence of cryosections (Dedek et al. 2006) concluded that Cx45 and Cx36 were never coexpressed in the same neuron, with the further assertion (Han and Massey 2005) that Cx45 and Cx36 proteins are never detected in the same fluorescent punctum. However, Dedek and coworkers reported that where Cx45 was present, it was present along with Cx36 in 30 % of puncta. Thus, those three groups separately concluded that because those cells are known to be coupled via gap junctions, their coupling required either (1) heterotypic coupling of Cx45 and Cx36 (i.e., that some additional factor allowed permissive coupling in vivo) or (2) that there must exist two additional connexin coupling partners to which Cx45 and Cx36 can separately couple.

In our initial FRIL studies of Cx36 vs. Cx45 in 671 double-labeled gap junctions in the inner plexiform layer of rat and mouse retina (Li et al. 2008a), single-replica FRIL showed that ca. 90 % (607) contained only Cx36, whereas ca. 9 % (58) contained both Cx45 and Cx36 in the same hemiplaques (and a statistically insignificant <1 %, mostly very small, contained minimal labeling for Cx45 without labeling for Cx36). Those data demonstrated conclusively that the 58 neurons with hemiplaques containing labeling for Cx45 and Cx36 must have synthesized both Cx45 and Cx36 proteins, conceivable at different times (thereby possibly accounting for failure to detect one or the other connexin), or alternatively, suggesting that the failure to simultaneously detect both connexins in any neurons represented a limitation of the detection method. Nevertheless, by single-replica FRIL, we were unable to ascertain whether the coupling partners of the double-labeled gap junction hemiplaques expressed either or both of those same connexins in the apposed hemiplaque. This question could be answered only by DR-FRIL, as developed and applied below.

In this article we discuss the advantages of matched DR-FRIL in identifying the cellular types contributing specific connexins to neuronal gap junctions in retina and goldfish hindbrain.

## Materials and Methods

**Fig. 3:**
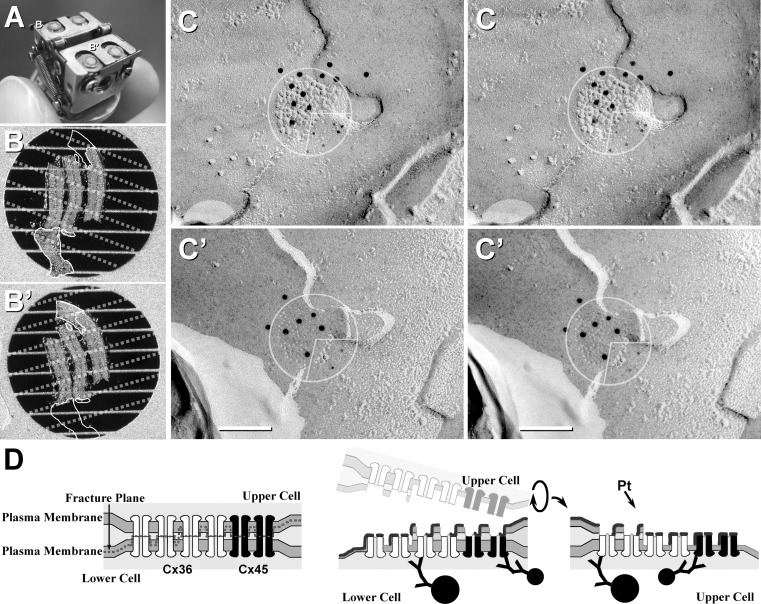
Explanation of the DR-FRIL technique. **a** Photograph of the DR stage after fracturing of two specimens. Matched DR samples (indicated by *B* and *B′* in **a**) are shown at higher magnification in **b**, **b′**), after floating off into buffer. **b**, **b′** Replicated but undigested samples were mounted on thin-bar grids, with matching outlines indicated. Small tissue fragments were lost during washing (*open outlines* opposite corresponding *outlined tissues*). Bars occluding matching areas are indicated by *dotted lines*. **c**, **c′** One of 11 pairs of matched gap junction hemiplaques (*circles with inscribed quadrants*) from the samples indicated in **b**, **b′**. Cx45 is labeled with 5-nm gold beads (*lower right quadrants*), whereas Cx36 is labeled with 10-nm gold beads (*upper left three quadrants* **c**, **c′**). Labeling for Cx45 is aligned with (i.e., opposite) labeling for Cx45 in the matching areas of the two hemiplaques. Likewise, labeling for Cx36 is aligned with labeling for Cx36. These matching hemiplaques demonstrate bihomotypic gap junctions. **b**, **c** Modified from Li et al. (2008a). **d** Diagram showing production of matched DRs and the subsequent immunogold labeling of bihomotypic plaques containing mostly Cx36 (*white connexons* labeled with *large gold beads*) and fewer Cx45 (*black connexons*, labeled with *small gold beads*)

Animals used in this study were prepared under protocols approved by the Institutional Animal Care and Use Committee of Colorado State University and conducted according to *Principles of Laboratory Animal Care* (U.S. National Institutes of Health publication 86-23, rev. 1985). These protocols included minimization of stress to animals and minimization of the number of animals used. One formaldehyde-fixed rat retina and several formaldehyde-fixed goldfish hindbrains were prepared for DR-SDS-FRL and DR-FRIL, as previously described (Li et al. 2008a; Pereda et al. 2003). Fixed tissues were sliced to 150 μm thickness at 4 °C in a refrigerated vibrating microslicer (DTK-1000; Dosaka, Kyoto, Japan) or a Lancer Vibratome 3000 (Leica Microsystems, Buffalo Grove, IL), infiltrated with 30 % glycerol as a cryoprotectant to reduce freezing damage by ice crystals, placed between two 4.6-mm gold planchettes and either frozen in a BalTec (BalTec, Balzers, Liechtenstein) 010 High-pressure Freezing Device (retina) or plunge-frozen in a mixture of 2:1 propane/ethane at −195 °C (hindbrain). Sandwich samples were placed in a prototype double-replica device (Fig. 3a), the two planchettes were mechanically separated (fractured) and the two newly created mirror complements were coated with 2–5 nm of carbon and shadowed with ~1.5 nm of platinum, thereby creating matched double-replicas (Li et al. 2008a). These samples were either thawed, with the tissues picked up on copper “thin-bar grids” and mapped by reflectance microscopy (Fig. 3b, b′, showing matching complements of adult rat retina), or bonded to a gold “index grid” [not shown but see Pereda et al. (2003)] before SDS washing and immunogold labeling. Both matched replica complements of retina were immungold-labeled simultaneously according to our previous descriptions (Li et al. 2008a), with 5-nm gold beads used to label Cx45 and 10-nm gold beads used to label Cx36. Matched replicas of goldfish hindbrain were labeled with monoclonal MAB3045 (Biosciences Research Reagents; Millipore, Temecula, CA) against Cx35 and polyclonal rabbit antibody against Cx34.7 [lot 2930-1 IL, from O’Brien et al. (2004)], with a single size of gold bead (5 nm) on goat anti-rabbit IgG for Cx45 and a different size of gold bead (10 nm) on goat anti-mouse IgG against Cx36.

## Results

### Procedures and Applications of DR-FRIL

We have previously described the method of FRIL and details of its applications (Rash et al. 2001; Rash and Yasumura 1999). DR-FRIL differs from FRIL in several respects. First, both sides of the fracture plane are retrieved and, not incidentally, may be matched by laborious repeated examination and matching of corresponding “fiduciary marks” that nevertheless may be difficult to recognize because they have opposite structural contour, or they may be covered by grid bars or damaged during SDS washing and labeling. Nevertheless, the rewards in obtaining definitive information regarding the connexin content, for example, of matched mirror complements are unequaled by any other technique. Equally important, a wide variety of scaffolding and accessory proteins can now be mapped and correlated with the biochemical composition and functional state of individual gap junctions in two apposed cells, as shown next.

#### DR-FRIL Reveals Connexin Coupling Partners in Retinal Gap Junctions

To determine whether Cx45-containing gap junctions are heterotypic vs. homotypic (and, hence, to identify connexin coupling partners in individual gap junctions), three cross sections of adult rat replica were prepared by DR-SDS-FRL and simultaneously double-labeled for Cx36 and Cx45. We found more than 160 gap junctions labeled for Cx36, 12 of which also contained Cx45. Eleven of those were found in the matched complementary replica (Fig. 3c, c′), and all 11 of the complements also had Cx45, along with Cx36. None were found with Cx45 alone, and no additional Cx45-labeled gap junctions were found in the other complement. This means that of 11 examples of Cx45-containing gap junctions encountered, all contained both Cx36 and Cx45 on both sides, thereby demonstrating that 100 % of those 22 coupled cells synthesized both Cx45 and Cx36. Moreover, the two connexins appeared to reside in separate domains, with labeling for Cx45 aligned with (i.e., was opposite) labeling for Cx45 in the matched hemiplaque and labeling for Cx36 aligned with labeling for Cx36 (Fig. 3, matching inscribed quadrants). Thus, these gap junctions were not heterotypic, as proposed by others, nor did either side need to contain additional unidentified connexins for establishing permissive connexon channels. Rather, those 11 gap junctions were bihomotypic, with Cx45 apparently coupling to Cx45 and Cx36 apparently coupling to Cx36. This overcame the problem of what had previously appeared to be heterotypic coupling between nonpermissive Cx45 and Cx36. Finally, the fortuitous segregation of connexin labels within gap junctions (rather than labels being completely intermixed) was consistent with suggestions that the connexons were in homomeric domains (i.e., each domain within an individual gap junction plaque contained only a single connexin type).

#### DR-FRIL Revealed Heterotypic Gap Junctions at Synapses on Neurons of Goldfish Hindbrain

Neurons in the tetrapod lineage have Cx36 as their primary connexin. In contrast, teleost fish duplicated their entire genomes from the parent vertebrate lineage, resulting in two homologs of mammalian Cx36, which diverged slightly as Cx34.7 and Cx35 (>85 % homology). These connexins vary primarily in their phosphorylation sequences [Cx34.7 lacks a phosphorylation site for CaMKII (Flores et al. 2010)] and in their membrane targeting sequences (O’Brien et al. 1998). In a first step toward investigating the presence of these two homologs in goldfish, we applied DR-FRIL to the analysis of gap junctions in a wide variety of neurons in the hindbrain.

**Fig. 4:**
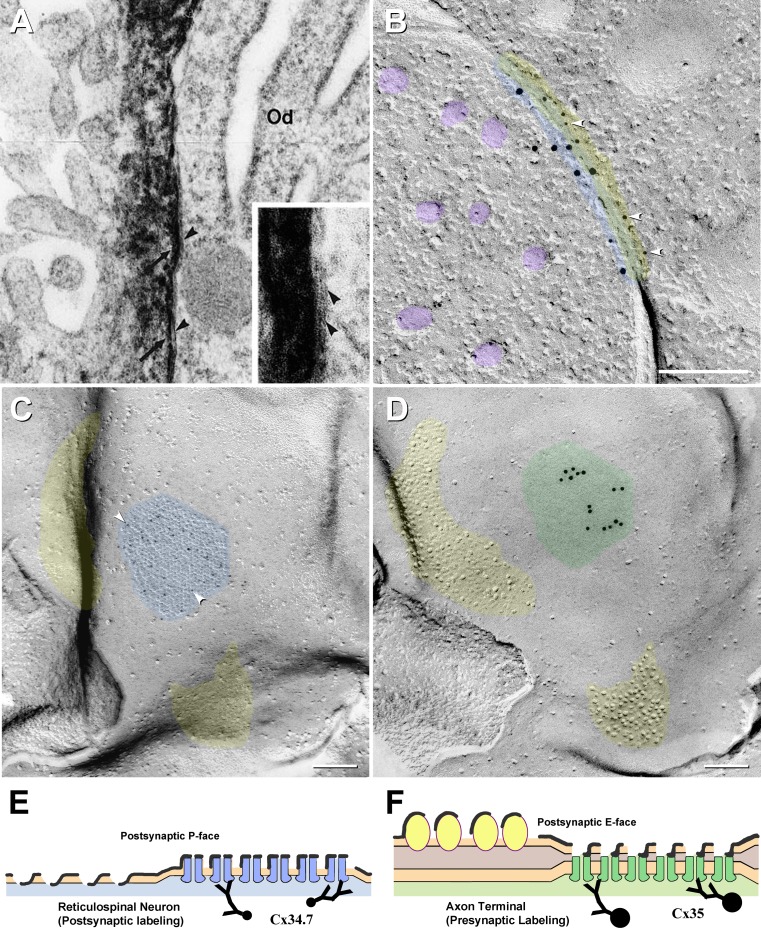
Comparison of heterotypic labeling in TEM thin sections (**a**), cross-fractured FRIL images (**b**) and by the DR-FRIL technique (**c**, **d**), with explanatory drawing (**e**, **f**). **a** Thin-section immunocytochemical demonstration of Cx43 in the astrocyte side of an O:A gap junction, labeled by the peroxidase–antiperoxidase method, leaving DAB deposition on the astrocyte side (*arrows*) and the oligodendrocyte side (*Od*) unlabeled (*arrowheads*). [This image was obtained 10 years before Cx47 was identified as the coupling partner for Cx43; modified from Ochalski et al. (1997)]. **b** Cross-fractured “mixed” (electrical plus chemical synapse), presumably from an auditory afferent onto an unidentified reticulospinal neuron in goldfish hindbrain. In this companion image to (**c)**, 5-nm gold beads labeled postsynaptic connexin Cx34.7 (*arrowheads*), whereas 10-nm gold beads for Cx35 labeled presynaptic connexins. (Synaptic vesicles are indicated by *purple overlays*.) The *yellow overlay* indicates the radius of uncertainty of immunogold labeling for small gold beads, the *blue overlay* indicates the radius of uncertainty for large gold beads and the *green overlay* indicates the region of potential overlap. This asymmetric distribution of gold labels reveals that this gap junction between a sensory afferent and the reticulospinal neuron is heterotypic. **c, d** Matching complementary replicas at club ending synapse on reticulospinal neuron. The postsynaptic hemiplaque (**c**, designated by *blue overlay*) is labeled for Cx34.7 by approximately three 5-nm gold beads (*arrowheads*), whereas the complementary E-face (*green overlay*) is labeled for Cx35 by 15 10-nm gold beads. Areas corresponding to glutamate receptor–containing postsynaptic densities are indicated by *yellow overlays*, with the P-face pits in **c** matching the E-face particles in (**b)**, as previously shown by labeling for glutamate receptors (Pereda et al. 2003). **e**, **f** Diagram of matching replica complements, showing Cx34.7 without Cx35 in the reticulospinal neuron (**e**) and Cx35 labeling without labeling for Cx34.7 in the E-face of the matching hemiplaque of the apposed club ending. Labeling in (**d**) and (**f**) is for connexins in the cytoplasm of the underlying axon terminal ending, even though only the E-face pits of the reticulospinal neuron are visualized in the replica

For comparison to our previous thin-section TEM images of heterotypic coupling at O:A gap junctions (Fig. 4a, inset), we show a rare cross-fractured gap junction with heterotypic labeling (Cx35 presynaptic and Cx34.7 postsynaptic, Fig. 4b) and matched DR complements of the an en face view of a gap junction on a reticulospinal neuron (Fig. 4c, d), with only small (5 nm) gold beads labeling postsynaptic connexins and only larger (10 nm) gold beads labeling presynaptic connexins. A diagram depicting the formation of a corresponding DR-FRIL replica is shown (Fig. 4e, f).

These preliminary data document expression of Cx34.7 without Cx35 in hemiplaques of a goldfish reticulospinal neuron, as well as Cx35 without Cx34.7 in the matching hemiplaques of its apposed axon terminal (Fig. 4c, d). In these matching complementary replicas, only Cx34.7 is detected in the reticulospinal neuron hemiplaque (Fig. 4c, arrowheads), whereas Cx35 is detected without Cx34.7 in the presynaptic hemiplaque that underlies the gap junction E-face pits (Fig. 4d). E-face pits are only faintly resolvable because of the 4- to 5-nm-thick carbon “precoat.” Although it is not yet determined whether the two types of coupled neurons (sensory and motor neurons) synthesize both connexins (with Cx35 targeted to axon terminals and Cx34.7 targeted to neuron soma and dendrites) or whether each neuronal subtype synthesizes only a single connexin isoform (Cx35 in sensory neurons and Cx34.7 in motor neurons), these data from DR-FRIL provide the molecular basis for a potential rectification of electrical transmission between these neurons.

Heterotypic gap junctions between neurons may contribute to the functional diversity of electrical transmission and provide a mechanism for preferred directionality of signaling (including electrical rectification), while bi- and trihomotypic gap junctions may provide for bidirectional signaling but with the added property of differential modulation of multiple conductance states for ions and small signaling molecules.

## Summary

DR-FRIL and DR-SDS-FRL have unambiguously revealed individual gap junctions expressing two connexins—on the one hand, forming bihomotypic and, on the other hand, forming heterotypic junctions. As a now widely recognized type of synapse between neurons in the mammalian CNS, the conductance properties of electrical synapses and their regulation take on new importance. The two different connexin distributions demonstrated by DR-FRIL (i.e., bihomotypic vs. heterotypic) suggest the existence of previously unrecognized mechanisms for regulating gap junction conductance states. Bi- and trihomotypic gap junctions are likely to increase the complexity and enhance the quality of synaptic communication provided by gap junctions. Likewise, heterotypic gap junctions provide not only for differential modulation on opposite sides of the same gap junction but also for the possibility of electrical rectification in teleost CNS neurons. Because there appears so far to be only one connexin (Cx36) widely expressed in neurons in tetrapods, heterotypic coupling may seem at first glance to preclude rectification. However, functional asymmetry between apposed hemichannels could perhaps be achieved by differences in posttranslational modifications between these two channels. Supporting this possibility, Cx36-containing junctions have been shown to coexist at different degrees of phosphorylation in the retina (Kothmann et al. 2009). If found in the mammalian CNS, electrical rectification and modulation of rectification would open up new pathways for synaptic communication that may be of particular relevance in the human CNS and in human neurological disease.

## Future Directions

As powerful as FRIL has proven to be, there are two limitations that make it and DR-FRIL less inviting for the novice. First, freeze-fracture and FRIL require complex, costly equipment that is at the cutting edge of high-vacuum, cryopreservation and metal evaporation technologies. Second, with more than a dozen discrete steps, all of which must be performed flawlessly to obtain the matching complementary replicas, DR-FRIL is particularly demanding of its technological practitioners. Third, freeze-fracture electron microscopy requires considerable time to gain expertise in obtaining and interpreting FRIL images. The training period for FRIL is several years because the researcher must learn conventional electron microscopy as well as interpretation of tissue ultrastructure in freeze-fracture replicas before learning FRIL methods. In these days of automated molecular biology, few students are willing to make this long-term commitment. With few laboratories remaining that are capable of conducting FRIL and DR-FRIL, there is a narrowing window of opportunity for the application of simultaneous ultrastructural and immunocytochemical approaches, not just to gap junctions and mixed synapses at EM resolution but also to other key questions in neuroscience requiring this level of resolution.

## Abbreviations

DR: Double replica
E-face: Extraplasmic leaflet
FRIL: Freeze-fracture replica immunogold labeling
P-face: Protoplasmic leafle
RT-PCR: Reverse transcriptase polymerase chain reaction
